# The Role of Microglia/Macrophages Activation and TLR4/NF-κB/MAPK Pathway in Distraction Spinal Cord Injury-Induced Inflammation

**DOI:** 10.3389/fncel.2022.926453

**Published:** 2022-06-09

**Authors:** Weishi Liang, Bo Han, Yong Hai, Yuzeng Liu, Xing Liu, Jincai Yang, Duan Sun, Peng Yin

**Affiliations:** ^1^Department of Orthopedic Surgery, Beijing Chaoyang Hospital, Capital Medical University, Beijing, China; ^2^Department of Pathology, Beijing Tiantan Hospital, Capital Medical University, Beijing, China

**Keywords:** distraction spinal cord injury, microglia/macrophages, neuroinflammation, TLR4 signaling pathway, porcine model

## Abstract

Distraction spinal cord injuries (DSCIs) often occur as the neurological complication of distraction forces following the implantation of internal fixation devices during scoliosis correction surgery. However, the underlying mechanism behind these injuries remains unclear. The present study aimed to explore the activation of microglia and macrophages, as well as changes in TLR4-mediated NF-κB and MAPK pathway activity after DSCIs in Bama miniature pigs. Prior to surgical intervention, the pigs were randomly divided into three groups: the sham group, the complete distraction spinal cord injury (CDSCI) group, and the incomplete distraction spinal cord injury (IDSCI) group. After surgery, the Tarlov scale and individual limb motor scale (ILMS) were used to evaluate changes in the pigs’ behavior. All pigs were euthanized 7 days after surgery, and histopathological examinations of the spinal cord tissues were performed. Immunohistochemistry was used to detect Caspase-3 expression in the anterior horn of spinal gray matter tissues. Immunofluorescence staining was utilized to assess the M1/M2 phenotype changes in microglia/macrophages and NF-κB P65 expression in central DSCI lesions, while western blotting was performed to determine the expression of TLR4/NF-κB/MAPK pathway-related proteins. The results of the present study showed that the Tarlov and ILMS scores decreased significantly in the two DSCI groups compared with the sham group. Hematoxylin and eosin (HE) and Nissl staining revealed that the tissue structure and nerve fiber tracts in the distracted spinal cord tissues were destroyed. Both DSCI groups showed the number of survived neurons decreased and the Caspase-3 expression increased. The results of the immunofluorescence staining indicated that the CD16 and CD206 expression in the microglia/macrophages increased. Between the two DSCI groups, the CDSCI group showed increased CD16 and decreased CD206 expression levels. The intensity of the fluorescence of NF-κB P65 was found to be significantly enhanced in pigs with DSCIs. Moreover, western blot results revealed that the expression of TLR4, p-IκBα, NF-κB P65, p-JNK, p-ERK, and p-P38 proteins increased in spinal cord tissues following DSCI. The present study was based on a porcine DSCI model that closely mimicked clinical DSCIs while clarifying DSCI-associated neuroinflammation mechanisms, in turn providing evidence for identifying potential anti-inflammatory targets.

## Introduction

Distraction spinal cord injuries (DSCIs) are the most common type of spinal cord injuries (SCIs), as well as one of the most severe surgical complications, in patients undergoing corrective surgery for scoliosis. Corrective surgery is the most effective treatment method for patients with scoliosis who have a Cobb angle >50° (Xie et al., 2012). During corrective surgery, distraction and compression on the concave side of the curve, along with excessive longitudinal stretching of the spine, are prone to causing DSCIs, resulting in motor-sensory and autonomic nerve dysfunction, and even paraplegia or quadriplegia (Shimizu et al., 2018). Studies have shown that the rate of SCIs in patients with scoliosis who have undergone corrective surgery with associated neurological monitoring was 0.8%, which produces a hefty socioeconomic load (Schwartz et al., 2007). The mature animal model types of SCIs being studied include spinal cord dislocation, contusion, and cutting, while there are few studies on DSCIs (Ju et al., 2014). The injury mechanism (apoptosis, inflammation, axonal degeneration, etc.) varies for different types of SCIs; therefore, it is necessary to conduct in-depth research on the injury itself, as well as potential treatment mechanisms, for DSCIs (Choo et al., 2008).

The pathophysiological process behind SCIs consists of a primary injury caused by mechanical factors (distraction, trauma, etc.), along with a secondary injury. Secondary SCIs are series of pathological reactions which occur within a few hours to several weeks after the initial SCI, further increasing the degree and scope of the injury (Alizadeh et al., 2019). Microglial activation and the release of proinflammatory cytokines are the primary manifestations of the inflammatory response during the secondary SCI period (Xu et al., 2021). In the SCI microenvironment, microglia/macrophages polarize into two phenotypes: a classically activated proinflammatory (M1) phenotype, and an alternatively activated anti-inflammatory (M2) phenotype. Additionally, M1 microglia/macrophages are quickly activated within the first 7 days, while fewer M2 microglia/macrophages are activated (Gaojian et al., 2020). Microglia and macrophages can release proinflammatory cytokines, such as tumor necrosis factor-α (TNF-α), interleukin-1 beta (IL-1β), and interleukin-6 (IL-6), which can directly promote neuronal death (An et al., 2021).

The Toll-like receptor 4 (TLR4) pathway is crucial in activated microglia-induced neuroinflammatory responses, and TLR4 is expressed with increased expression levels on the microglial membrane after an SCI (Reed-Geaghan et al., 2009; Impellizzeri et al., 2015). In the central nervous system, Lipopolysaccharide (LPS) binds to and activates TLR4, leading to the activation of nuclear factor kappa B (NF-κB) and mitogen-activated protein kinases (MAPKs; Lee and Lee, 2002). The MAPK pathway includes P38 MAPK, c-Jun N-terminal kinase (JNK), and extracellular signal-related kinases (ERK), which are phosphorylated during the inflammatory response to promote the production of inflammatory mediators (Rahimifard et al., 2017). Many studies have shown that the TLR4-mediated activation of NF-κB and MAPK in microglia after an SCI is an important mechanism of inflammatory injury in spinal cord tissue; however, its role in DSCIs remains unclear (Impellizzeri et al., 2015; Kasuya et al., 2018; Xu et al., 2018). Therefore, these two pathways are of great significance in studying the mechanism of inflammatory injuries and identifying potential therapeutic targets after DSCIs.

In a previous study, we successfully established DSCI porcine models and found that the expression of the microglial marker Iba-1, as well as the proinflammatory cytokines TNF-α, IL-1β, and IL-6, increased after DSCIs (Han et al., 2022). In the present study, we used a Bama miniature pig DSCI model as the research object, and further explored the activation of microglia and macrophages, as well as the changes in TLR4-mediated NF-κB and MAPK pathway activity after DSCI, to provide evidence for finding potential anti-inflammatory targets for these injuries.

## Materials and Methods

### Animal Caring

The animal experiment protocols were approved by the Medical Ethics Committee of Capital Medical University (AEEI-2019-098), China. We purchased SPF Bama miniature pigs aged 3 months (*n* = 9, 11.40 ± 1.68 kg, China) to establish DSCI animal models and adaptatively fed them for 1 week. These pigs were housed and underwent surgery in the Experimental Animal Center of Capital Medical University. These animals were maintained in environmentally controlled rooms with a 12:12-h light: dark cycle in isolation units. All experimental procedures referred to animals complied with the Guide for the Care and Use of Laboratory Animals published by the National Institutes of Health (NIH Publication No. 8523, revised 2011, United States).

### Animal Grouping and Intraoperative Neuromonitoring

**FIGURE 1:**
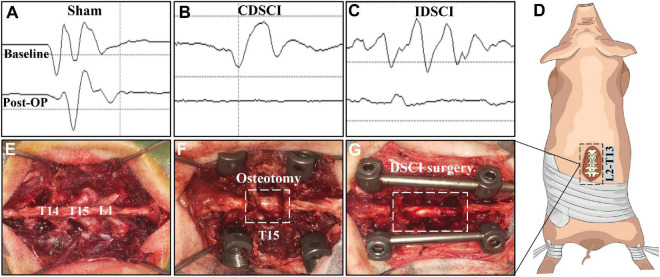
The surgical procedure of the DSCI model establishment and the intraoperative MEP changes in different groups. **(A–C)** Typical MEP amplitude changes of the pigs in the sham, CDSCI, and IDSCI groups were shown. **(D)** The intraoperative incision was T13-L2. **(E)** T14, T15, and L1 laminae were exposed using coagulation cautery. **(F)** The pedicle screws were inserted in T14 and L1, and the T15 vertebral body and adjacent intervertebral discs were completely resected. **(G)** The DSCI porcine models were successfully established by gradually increasing the interval between the T14 and L1 using a spinal spreader.

**FIGURE 2:**
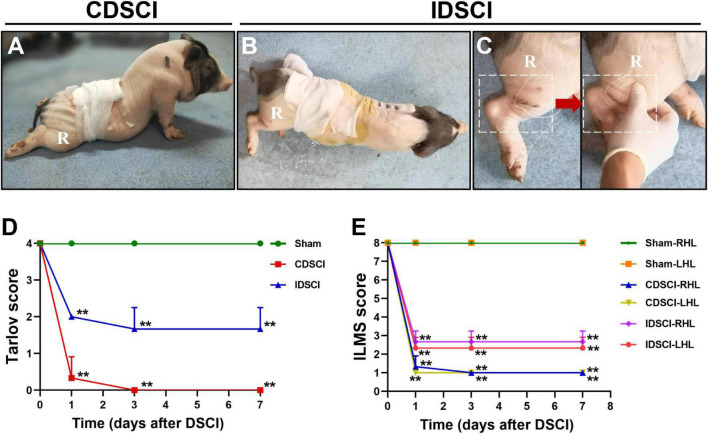
The neurologic function changes in the hind limbs after DSCI. **(A,B)** The walking gait of pigs in the CDSCI and IDSCI groups was shown. **(C)** Muscle contraction was observed in the porcine hind limbs by stimulating the muscles in the IDSCI group. **(D,E)** The Tarlov and ILMS scale were used to functionally score the pigs of the sham, IDSCI, and CDSCI groups at the day 1, 3, and 7 post-surgery (*n* = 3). All values are expressed as means ± SD. *P < 0.05, **P < 0.01, vs. the sham group.

**FIGURE 3:**
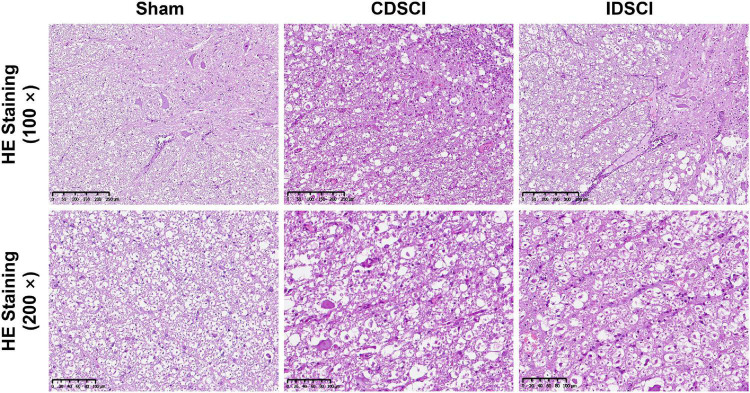
The pathological changes in the spinal cord tissues of different groups after DSCI. The representative images of spinal cord tissues stained with HE staining (scale bar = 200 μm or 100 μm) were shown.

All pigs were randomly assigned into three groups (*n* = 3 per group). (1) Sham group: pigs underwent T15 osteotomy and pedicle screw fixation with normal motor evoked potential (MEP; Figure 1A); (2) complete distraction spinal cord injury (CDSCI) group: pigs underwent T15 osteotomy plus DSCI surgery, with MEP amplitude decreased by 100% (Figure 1B); (3) incomplete distraction spinal cord injury (IDSCI) group: pigs underwent T15 osteotomy plus DSCI surgery, with MEP amplitude decreased by approximately 75% (Figure 1C). All pigs were sacrificed 7 days after surgery, and porcine spinal cord tissues were harvested and temporarily stored in 4% paraformaldehyde or liquid nitrogen for further experimental tests.

During the operation, we used an electrophysiological monitor (Cadwell, United States) to monitor MEP changes, which can reveal the occurrence of DSCI. The stimulating electrodes were inserted into the C3–C4 cortical motor area with a 100–200 V single stimulation intensity, while the recording and reference electrodes were inserted into the gastrocnemius muscles of the hind limbs. After anesthesia, the baseline of MEP baseline was established as a reference. MEP stimulation was performed at multiple surgical nodes, such as screw placement, osteotomy, and spinal distraction throughout the operation. Multipoint monitoring throughout the operation can also exclude other iatrogenic factors causing SCI.

### Establishment of Porcine Distraction Spinal Cord Injury Model

The surgical procedure for the DSCI model in Bama miniature pigs was consistent with that in our previous studies (Han et al., 2022). After anesthetizing the animals, a midline longitudinal incision was made extending from thoracic vertebra 13 (T13) to lumbar vertebra 2 (L2; Figure 1D). The laminae from T14 to L1 were exposed, and temporary fixation was placed by inserting short caudal pedicle screws (4.0 mm × 25 mm, Weigao, Shandong, China) and SINO rods into T14 and L1 (Figures 1E,F). Next, a global column osteotomy was performed at T15, and the adjacent intervertebral discs were resected. Finally, a spreader was used to gradually increase the space between T14 and L1 by 1 mm (Figure 1G). When the motor evoked potential (MEP) amplitude decreased by 100%, or approximately 75%, distraction was stopped, and the amplitude change was maintained for 10 min. Cefuroxime sodium was administered to prevent infection during the first 3 days after surgery, and the dressing was changed every 3 days. Additionally, each pig’s bladder was manually emptied twice a day, while rigorous care of the perineum was performed on each pig.

### Anesthesia Monitoring

All pigs were fasted for 24 h prior to anesthesia. The pigs were first given 3% pentobarbital sodium solution by intramuscular injection, and the operation was performed under continuous inhalation anesthesia with isoflurane. Each pig was intubated with an endotracheal tube, and the depth of anesthesia was monitored using pain and corneal reflex test. At the same time, using a multifunctional bedside physiological monitor (Nihon Kohden, Japan) to monitor the vital signs of the pigs.

### Postoperative Behavior Assessment

To evaluate the success of the DSCI modeling and associated changes in the nerve function of the pigs after surgery, we assessed the individual motor function of the bilateral limbs. Behavioral assessments were performed using the traditional Tarlov scale (Aslan et al., 2009; Table 1) and the individual limb motor scale (ILMS; Cerro et al., 2021; Table 2). Tarlov scores were used to evaluate the movement, weight-bearing, and walking abilities of the hind limbs, whereas the ILMS scores primarily assessed the muscle strength, limb positioning, joint angles, weight support, and walk stepping of each hind limb. All animals were assessed with both scales on post-surgery days 1, 3, and 7.

**TABLE 1:** Tarlov scale.

Score	Description
0	Complete paralysis of the lower limbs
1	Slight movement of lower limbs
2	Good movement of the lower limbs but unable to stand
3	Able to stand but unable to walk
4	Complete recovery of motor function and walk normally

**TABLE 2:** Individual limb motor scale (ILMS).

Score	Description
1	Flaccid, inactive limb
2	Weak and irregular limb movement, no weight bearing
3	Robust and regular movement in one or more joints, abnormal joint angles and limb positioning, no weight bearing
4	Normal positioning of the limb, partial weight support, abnormal joint angles
5	No visible impairment for joint flexion and extension, partial weight support
6	Complete body weight support for standing but not for stepping
7	Apparently normal stepping with mistakes in limb coordination
8	Apparently normal use of the limb

### Hematoxylin and Eosin Staining and Nissl Staining of Spinal Cord Tissues

The spinal cord samples in the central DSCI lesions were acquired after sacrifice and were fixed in 4% paraformaldehyde for 24 h. Then these samples were undergone conventional paraffin embedding and were sliced into 4 μm-thick slices using a slicer. After 4-h roasting at 60°C, the paraffin sections were dewaxed, hydrated, and stained using an hematoxylin and eosin (HE) Staining Kit (Solarbio, Beijing, China) and Nissl Staining Kit (Solarbio) according to the manufacturer’s instructions, respectively. The stained sections were scanned and visualized using a NanoZoomer S60 digital slide scanner (Hamamatsu, Japan) and NDP.view2 viewing software (Hamamatsu, Japan), respectively.

### Immunohistochemistry

Spinal cord tissue samples in 4 μm thick paraffin sections were dewaxed, hydrated, and boiled to repair the antigen. After washing with phosphate-buffered saline (PBS), the sections were soaked in goat serum and then incubated overnight at 4°C with the following primary antibodies: anti-Caspase-3 (1:75, Abcam). The sections were subsequently incubated with horseradish peroxidase (HRP)-conjugated goat anti-rabbit or goat anti-mouse IgG secondary antibodies (1:500, Solarbio) at room temperature for 1 h. After washing with PBS, the sections were stained with 3,3′-Diaminobenzidine (DAB) chromogenic fluid, and nuclei were stained with hematoxylin for 2 min. Finally, the expression of positive proteins was visualized using the NDP.view2 viewing software. The integrated optical density of staining in three representative fields for each section (one section per pig, three pigs per group) was analyzed using Image-Pro Plus software (Media Cybernetics, United States).

### Immunofluorescence Staining

Spinal cord paraffin sections (4 μm thick) from each specimen were dewaxed, hydrated, and boiled to repair the antigen. Sections were blocked with 0.1% Triton X-100 and 10% normal goat serum at room temperature for 2 h. Sections were incubated overnight at 4°C with the following primary antibodies: anti-CD16 (1:200; Bioss), anti-CD206 (1:5000; Proteintech), anti-Iba-1 (1:200; Proteintech), and anti-NF-κB P65 (1:200; CST). The sections were rinsed 3 times using PBS, 5 min each time, after which the sections were incubated with secondary antibodies for 1 h at room temperature. Following the PBS rinsing, the nuclei were stained blue with 4′,6-diamidino-2-phenylindole (DAPI) (Solarbio). The sections were sealed with an anti-fluorescence quenching agent after processing, and immunofluorescence imaging was performed using a digital slide scanner. ImageJ software (National Institutes of Health, United States) was utilized to measure the mean fluorescence intensity of specific proteins.

### Western Blotting

The spinal cord samples were kept at −80°C until manually homogenized and added to RIPA lysis and extraction buffer. The supernatant was obtained after centrifugation at a low temperature, and the protein concentration was determined using the BCA method. Proteins were separated by SDS-PAGE and electrophoretically transferred to PVDF membranes. PVDF membranes were blocked with 5% non-fat dry milk for 2 h and subsequently incubated with primary antibodies overnight at 4°C. The primary antibodies used were as follows: anti-TLR4 (1:1000, GeneTex), anti-p-IκBα (1:1000, CST), anti-NF-κB P65 (1:1000, CST), anti-p-JNK (1:1000, CST), anti-p-ERK (1:2000, CST), anti-p-P38 (1:1000, CST), and anti-β-actin (1:5000, Proteintech). Then the membranes were incubated for 1 h with goat anti-rabbit or goat anti-mouse IgG HRP-conjugated secondary antibody (1:5000). At last, PVDF membranes were exposed using a Tanon 5200 chemiluminescence image analysis system. Following exposure, ImageJ software was used to analyze the gray values of bands.

### Statistical Analysis

All data are presented as mean ± standard deviation (SD). Measurements were compared with Student’s *t*-test or one-way analysis of variance (ANOVA) using GraphPad Prism software (United States) or SPSS 22.0 (United States). A Bonferroni adjustment was carried out for multiple comparisons of the three groups. Results were considered statistically significant at *P < 0.05, and ^**^P < 0.01 when comparing the sham group; ^#^*P* < 0.05, ^##^*P* < 0.01 when comparing the CDSCI group.

## Results

### Neurologic Function Changes of Hind Limbs After Distraction Spinal Cord Injury

The pigs in the CDSCI and IDSCI groups showed irreversible neurological damage 7 days after surgery. The hind limbs of CDSCI pigs were completely paralyzed (Figure 2A); the muscle strength of the hind limbs in the IDSCI group was significantly decreased, and weak and irregular limb movement occurred when the hind limbs were stimulated by squeezing (Figures 2B,C). The assessment results of the Tarlov score and ILMS score at the 1, 3, and 7 days after DSCI was presented in Figures 2D,E. Compared with the sham group, the Tarlov score of the two DSCI groups decreased significantly at each time point (all *P* < 0.01), while the CDSCI tends to 0. For the ILMS score, the neurological function of the left and right hind limbs of the CDSCI and IDSCI groups was compared with the corresponding side of the sham group. At each time point, the ILMS score of both sides of the hind limbs in the two DSCI groups showed a significant decrease when compared with the sham group (all *P* < 0.01). And the decrease in the CDSCI group is more significant.

### Pathological Changes in the Spinal Cord Tissues After Distraction Spinal Cord Injury

As shown in Figure 3, HE staining was used to visualize pathological changes in the spinal cord. In the sham group, the structure of the spinal cord tissue was normal, the nerve fibers and connective tissue in the white matter were arranged regularly, and the neurons were normal and abundant. Evaluation of the spinal cords that underwent DSCI surgery revealed disorganized white and gray matter structures, obvious bleeding, increased inflammatory cell infiltration, decreased nerve fiber density, and edema of the myelin sheath. The number of neurons and neurites decreased significantly after the DSCI surgery. All of these pathological changes were more severe in the CDSCI than the IDSCI group.

### Survival of Neurons and Apoptosis Level in the Anterior Horn of Spinal Gray Matter After Distraction Spinal Cord Injury

**FIGURE 4:**
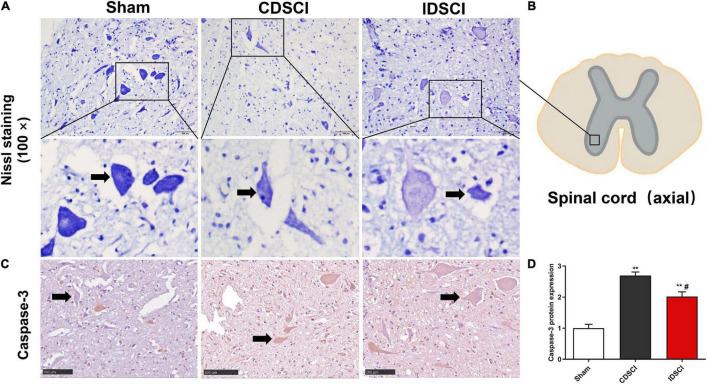
The survival of neurons (black arrows) and apoptosis level in the anterior horn of spinal gray matter tissues after DSCI. **(A)** Representative images of Nissl staining of neurons in the anterior horn of spinal gray matter tissues in the different groups. **(B)** The boxed area in the axial spinal cord diagram showed the approximate position of the captured images. **(C)** The representative immunohistochemical images regarding Caspase-3 of different groups are listed (scale bar = 100 μm). **(D)** The mean optical density (MOD = IOD/Area) of the immunohistochemistry-positive sites in the sample sections was determined as the protein expression level (*n* = 3). All values are expressed as means ± SD. *P < 0.05, **P < 0.01, vs. the sham group; ^#^*P* < 0.05, ^##^*P* < 0.01, vs. the CDSCI group.

Nissl staining (Figures 4A,B) revealed that specimens from the anterior horn of the spinal gray matter in the sham group showed a normal shape with more neurites and deeper blue-stained Nissl bodies in the cytoplasm. In the two DSCI groups, the number of surviving neurons was significantly reduced, the cytoplasm was atrophied, and the number of Nissl bodies was decreased compared with the sham group.

Moreover, the immunohistochemical results showed that compared with the sham group, Caspase-3 expression was increased in anterior horn of the spinal gray matter tissues of the CDSCI and IDSCI group (*P* < 0.01), and the expression level of Caspase-3 was higher in the CDSCI group (*P* < 0.05) (Figures 4C,D).

### Expression Levels of M1/M2 Microglia/Macrophage Polarization Markers in the Spinal Cord Tissues After Distraction Spinal Cord Injury

**FIGURE 5:**
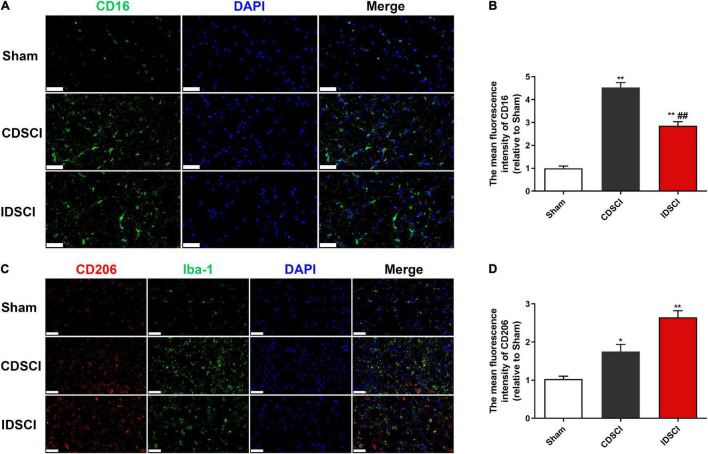
Expression levels of M1/M2 microglia/macrophage polarization markers in the spinal cord tissues after DSCI. **(A,C)** Representative immunostaining images of CD16 (green), CD206 (red), Iba-1 (green) and DAPI (blue) in the spinal cord tissues at day 7 post-surgery (scale bar = 50 μm). **(B,D)** The mean fluorescence density of CD16 and CD206 quantification in the aim field was measured (*n* = 3). All values are expressed as means ± SD. *P < 0.05, **P < 0.01 vs. the sham group; ^#^*P* < 0.05, ^##^*P* < 0.01, vs. the CDSCI group.

To determine if the DSCI affects the polarization state of microglia, immunofluorescence staining was used to detect microglia polarization markers. The results showed that the expression of M1 phenotype maker (CD16) and M2 phenotype maker (CD206) of microglia/macrophage was significantly increased in the two DSCI groups, compared with the sham group (*P* < 0.05) (Figure 5). For the comparison between the CDSCI and IDSCI groups, a significant increase in CD16 expression (*P* < 0.01) and a decrease of CD206 expression with no significant statistical difference (*P* > 0.05) were detected in the CDSCI group.

### Expression Levels of TLR4/NF-κB Signaling Pathway-Related Proteins in the Spinal Cord Tissues After Distraction Spinal Cord Injury

**FIGURE 6:**
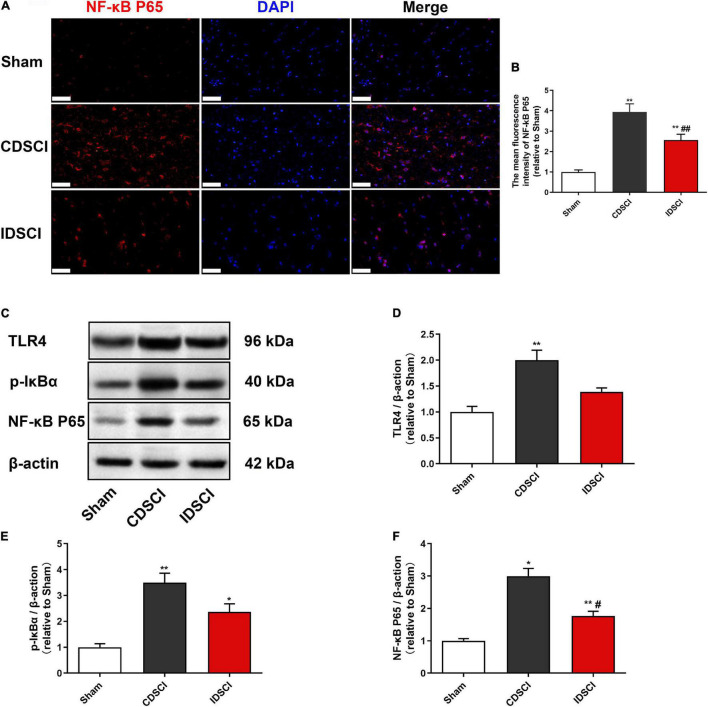
Determination of the expression levels of TLR4/NF-κB signaling pathway-related proteins in the spinal cord tissues after DSCI. **(A)** Representative immunostaining images of NF-κB P65 (red) and DAPI (blue) in the spinal cord tissues at day 7 post-surgery (scale bar = 50 μm). **(B)** The mean fluorescence density of NF-κB P65 quantification in the aim field was measured (*n* = 3). **(C)** The representative immunoblots of TLR4, p-IκBα, NF-κB P65, and β-actin are listed. **(D–F)** Grayscale values of TLR4, p-IκBα, and NF-κB P65 were determined using ImageJ software based on the bands in the immunoblots (*n* = 3). All values are expressed as means ± SD. *P < 0.05, **P < 0.01, vs. the sham group; ^#^*P* < 0.05, ^##^*P* < 0.01, vs. the CDSCI group.

To assess the expression of critical inflammatory protein NF-κB, we conducted immunofluorescence staining to detect its expression level (Figures 6A,B). The immunostaining result showed a significant increase in NF-κB P65 expression in the CDSCI and IDSCI groups compared with the sham group (*P* < 0.01). And the fluorescence expression level of the NF-κB P65 was higher in the CDSCI group between the two DSCI groups (*P* < 0.01).

Besides, western blot was used to detect TLR4, p-IκBα, and NF-κB P65 in the spinal cord tissues of different groups (Figures 6C–F). In comparison with the sham group, DSCI significantly increased the expression level of TLR4, p-IκBα, and NF-κB P65 in the spinal cord tissues of Bama pigs (*P* < 0.05), except the TLR4 of the IDSCI group with no significant statistical difference (*P* > 0.05). Compared with the CDSCI group, the IDSCI group showed a lower expression level of the TLR4, p-IκBα, and NF-κB P65 proteins in the spinal cord tissues, but no significant statistical difference was found in the TLR4 and p-IκBα (*P* > 0.05).

### Expression Levels of MAPK Signaling Pathway-Related Proteins in the Spinal Cord Tissues After Distraction Spinal Cord Injury

**FIGURE 7:**
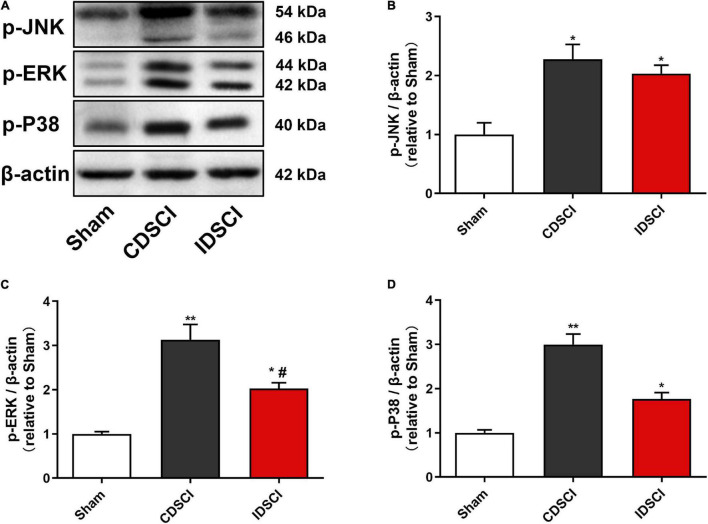
Determination of the expression levels of MAPK signaling pathway-related proteins in the spinal cord tissues after DSCI. **(A)** The representative immunoblots of p-JNK, p-ERK, p-P38, and β-actin are listed. **(B–D)** Grayscale values of p-JNK, p-ERK, p-P38 were determined using ImageJ software based on the bands in the immunoblots (*n* = 3). All values are expressed as means ± SD. *P < 0.05, **P < 0.01, vs. the sham group; ^#^*P* < 0.05, ^##^*P* < 0.01, vs. the CDSCI group.

As represented in Figure 7, the phosphorylation activation of the JNK/ERK/P38 signaling pathway was detected by western blotting. Compared with the sham group, the expression levels of p-JNK, p-ERK, and p-P38 proteins were significantly increased in the two DSCI groups (*P* < 0.05). For the two DSCI groups, there was a higher expression level of these three proteins in the CDSCI group, but no significant statistical difference was found for the p-JNK and p-P38 (*P* > 0.05).

## Discussion

**FIGURE 8:**
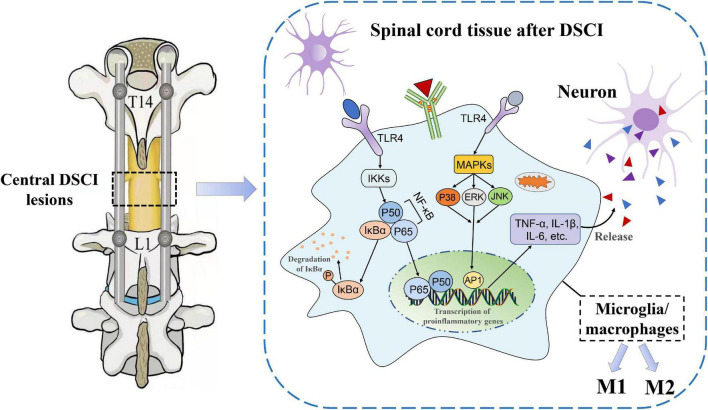
The underlying mechanisms of neuroinflammatory mechanisms in the spinal cord tissues after DSCI. In the DSCI microenvironment, microglia/macrophages were activated and polarized into proinflammatory M1 phenotype and anti-inflammatory M2 phenotype. The TLR4 pathway is activated after DSCI and promotes inflammatory response. On the one hand, TLR4 phosphorylates IκBα through the IKKs, rendering the NF-κB complex dimer (mostly p65/p50 dimers) free to translocate to the nucleus. On the other hand, the MAPK pathway, including P38 MAPK, JNK, and ERK, is phosphorylated by TLR4 during the inflammatory response, thereby activating activator protein (AP)-1. Nuclear NF-κB and AP-1 promote transcription of proinflammatory genes such as TNF-α, IL-1β, and IL-6 to exacerbate the neuroinflammatory reaction.

It is well known that neuroinflammation contributes to increased aggravation in the primary lesions and a decrease in nerve function from the secondary injury after an SCI. The release of proinflammatory cytokines after the activation of microglia and macrophages is directly related to the level of inflammation, which is the key cause of neuronal apoptosis and spinal cord tissue destruction (Papa et al., 2013). The results of our previous study (Han et al., 2022) showed that IL-1β, IL-6, and TNF-α levels increased in the spinal cord tissue of pigs after being subjected to a DSCI, indicating a significant transformation of the injured spinal cord tissue to a pro-inflammatory environment. In the present study, neurological dysfunction did not heal in the Bama miniature pigs within the first 7 days after the DSCI. The results showed that microglia were significantly activated after the DSCI, and the M1/M2 phenotype of the microglia/macrophages increased with the aggravation of the DSCI, whereas the M2 phenotype decreased. It is possible that the TLR4-mediated activation of the NF-κB and MAPK pathways may contribute to M1 microglia-induced exacerbation of neuronal apoptosis (Figure 8).

Compared with other types of SCIs, the DSCI animal model is more challenging to establish and is primarily divided into acute and continuous DSCIs. Unfortunately, the distraction devices used for the construction of various animal models are not uniform. Most scholars have used self-made spinal distractors to create DSCI models in small animals (such as rats and rabbits) by briefly putting traction on the vertebral bodies on both sides of the distraction center in opposite directions (Seifert et al., 2011; Wu et al., 2016). Although these self-made devices can cause secondary inflammation and oxidative stress injuries to the spinal cord, they cannot achieve a continuous DSCI state. Continuous distraction of the spinal cord is a good choice for simulating iatrogenic DSCIs which occur during corrective surgery for scoliosis. Hong et al. (2016) performed surgery in pigs which involved a T13 full-column osteotomy plus spinal distraction, and the disappearance of the MEP signal was taken as the endpoint of distraction. The final distraction distance was found to be 74.3% (68–84%) of the segmental vertebral height. The technique used in our previous study (Han et al., 2022), involving a T15 global column osteotomy plus pedicle screw fixation, was also utilized in the present study to successfully establish the DSCI model, for which the Tarlov and ILMS scores showed significant and stable neurological function defects after DSCI. The method of stable and repeatable large-animal model establishment used in the present study is a reliable guarantee for subsequent experiments.

Because the distraction force of the spinal cord in DSCIs is parallel to the long axis of the longitudinal tension, the resulting microstructural damage has certain characteristics. Under an electron microscope, parts of the axons and blood vessels were found to be broken by the distraction force, white matter arrangement and structure were found to be disorganized, and the nuclei and organelles in neurons showed signs of early necrosis (Wu et al., 2016). The tissue damage characteristics of DSCIs observed under a light microscope were similar to those of other types of traumatic SCI. In the present study, HE staining of DSCI tissues revealed a disorganized white and gray matter structure, obvious bleeding, increased inflammatory cell infiltration, and significant myelin sheath vacuolation. Additionally, neurons in the gray matter showed apoptosis and necrosis, abnormal cell morphology, and decreased cell numbers. We did, however, observe the survival of motor neurons in the anterior horn of the spinal gray matter via Nissl staining, indicating that the number of surviving neurons decreased significantly in the two DSCI groups compared with the sham group. It was observed that increased elongation resulted in a decreased number of surviving neurons. Caspase-3 is a critical protease that is involved in apoptosis, and cytochrome C release and Caspase-9 activation can significantly increase within half an hour after an SCI, which is the upstream mechanism of Caspase-3 activation (Springer et al., 1999). The activation of Caspase-3 can affect Ca^2+^ regulation through the endoplasmic reticulum, in turn inducing an oxidative stress reaction and initiating and executing neuronal apoptosis (Pinton et al., 2008). In the present study, immunohistochemistry analysis showed that the expression level of Caspase-3 in the anterior horn of the spinal gray matter was significantly increased after DSCI, while the level of apoptosis was increased. These results indicate that DSCIs can damage the tissue structure and microstructure of the spinal cord, accelerate apoptosis and necrosis of motor anterior horn neurons, and lead to neurological impairment.

To evaluate the process of inflammatory damage *in vivo* at an early stage, experimental pigs were euthanized 7 days after surgery in the present study. It is widely known that the pro-inflammatory M1 and anti-inflammatory M2 phenotypes of microglia play a bidirectional role in the secondary injury stage of SCIs (Orihuela et al., 2016). M1 microglia are primarily activated and persist for a long time, after an SCI, leading to a cascade of inflammatory reactions (Wang et al., 2015). Previous studies have found that M1 microglia/macrophages can induce the apoptosis of neurons and oligodendrocytes, which can aggravate the destruction of nerve function (Kroner et al., 2014; Fan et al., 2016). These two activated types of microglia/macrophages can be discriminated by specific markers on their cell surface, such as CD16, inducible nitrous oxide synthase (iNOS) (in M1 macrophages), or CD206 and Arg-1 (in M2 macrophages). In the present study, immunofluorescence staining showed M1 and M2 microglia phenotypic markers (CD16 and CD206, respectively) in both DSCI groups at 7 days after the induction of the DSCI. For the two DSCI groups, the M1 phenotype increased, while the M2 phenotype decreased, with an increase in the degree of distraction. Additionally, it has been reported that applying the therapeutic intervention method to transform microglia from the M1 to the M2 phenotype after an SCI can promote behavioral function recovery, which may be a promising therapeutic mechanism (Liu et al., 2020). Another study showed that utilizing pharmaceuticals to facilitate the shift from M1 to M2 microglia/macrophage polarization may facilitate axon regeneration, increase myelin sheath reconstruction, reduce chondroitin sulfate formation, and inhibit scar hyperplasia (Gaojian et al., 2020). Therefore, future studies on the treatment of DSCIs and the reduction of spinal cord inflammation should focus on the conversion of the M1 to the M2 phenotype.

The inflammatory environment which occurs after an SCI promotes the activation and proliferation of microglia/macrophages and the release of proinflammatory cytokines, as well as other toxic mediators, which is closely related to neuronal apoptosis and neurite injury (Guadagno et al., 2013). The results of our previous study confirmed that TNF-α, IL-1β, and IL-6 levels increased significantly in damaged spinal cord tissue and cerebrospinal fluid after DSCI, while the survival rate of neurons decreased (Han et al., 2022). After undergoing mechanical stimulation such as distraction, the expression of TLRs in activated microglia of the impaired spinal cord tissue was significantly increased (Carpentier et al., 2008). A large number of TLRs are activated after DSCIs, of which TLR4 plays the most significant role in the inflammatory response, and can be further bound and stimulated by secreted cytokines (Chen et al., 2017). The activation of TLR4 promotes microglial polarization, which initiates the neuroinflammatory response after an SCI (Xu et al., 2020). Additionally, necroptosis of astrocytes can be partially induced by M1 microglia/macrophages through TLR4 signaling, which exacerbates secondary DSCIs (Fan et al., 2016). TLR4 can further phosphorylate IκBα through the inhibitory-κB kinase (IKK) complex, thereby releasing NF-κB for translocation to the nucleus and increasing the transcription of proinflammatory genes (i.e., TNF-α, IL-1β, an IL-6, among others) (Lotze and Tracey, 2005). In the present study, the expression levels of TLR4 and NF-κB P65, as well as the phosphorylation level of IκBα, significantly increased after DSCI, suggesting that the TLR4/NF-κB pathway plays an important role in microglia-mediated inflammation following DSCI.

Additionally, the activation of the MAPK pathway by ligand binding TLR4 is another important mechanism of SCI inflammation. In the MAPK pathway, ERK, JNK, and P38 proteins are phosphorylated to promote the release of proinflammatory factors (Ko et al., 2019). It was (Xu et al., 2006) reported that SCIs might activate microglia and macrophages by phosphorylating ERK1/2 and P38 MAPK proteins, the activation of which can initiate a sequence of iNOS-mediated cell death reactions. Through *in vitro* experiments, Shen et al. (2006) used LPS to intervene with microglia and found that the TLR4 signaling pathway increased the phosphorylation of JNK, P38 MAPK, and ERK, and also promoted the expression of proinflammatory cytokines and chemokines. In the present study, western blotting showed that p-JNK, p-ERK, and p-P38 levels were significantly increased after DSCI, which was consistent with the increased inflammatory cytokine levels of IL-1β, IL-6, and TNF-α. The results of another study showed that the inhibition of P38 MAPK activity inhibits NF-κB activity, suggesting that P38 MAPK has a positive regulatory effect on NF-κB activity in the inflammatory response (Jeong et al., 2014). Therefore, TLR4-mediated NF-κB and MAPK pathways may jointly respond to increased inflammation in secondary SCIs.

## Conclusion

The results of the present study indicated that continuous mechanical distraction of the spinal cord in Bama miniature pigs resulted in decreased neurological function, histopathological lesions, and neuronal apoptosis, which increased in severity as the degree of the DSCI increased. Study results regarding the mechanism behind DSCIs suggested that the activation of M1/M2 microglia and macrophages increased during the early period of the DSCI. When comparing the two DSCI groups, the CDSCI group has increased CD16 and decreased CD206 expression levels. TLR4-mediated NF-κB and MAPK pathways were found to be activated and potentially involved in the regulation of macrophage/microglia-mediated inflammation. These findings provide a reference for further clarifying the mechanisms of DSCI inflammatory injuries and developing new drugs targeting the injury mechanisms of microglia/macrophages inflammation.

## Data Availability Statement

The original contributions presented in this study are included in the article/supplementary material, further inquiries can be directed to the corresponding authors.

## Ethics Statement

The animal experiment protocols were approved by the Medical Ethics Committee of Capital Medical University (AEEI-2019-098).

## Author Contributions

YH and PY designed and supervised the study. YH, PY, YL, and JY performed the surgery to establish the experimental animal models. WL and BH participated in the research design and preparation and performed animal feeding, experimental tests, and statistical analysis. WL wrote the first draft of the manuscript. YH, PY, BH, YL, and JY critically revised the work. All authors read and approved the final version of the manuscript.

## Conflict of Interest

The authors declare that the research was conducted in the absence of any commercial or financial relationships that could be construed as a potential conflict of interest. The reviewer BB declared a shared parent affiliation with the authors to the handling editor at the time of review.

## Publisher’s Note

All claims expressed in this article are solely those of the authors and do not necessarily represent those of their affiliated organizations, or those of the publisher, the editors and the reviewers. Any product that may be evaluated in this article, or claim that may be made by its manufacturer, is not guaranteed or endorsed by the publisher.
